# A Peptide Nucleic Acid against MicroRNA miR-145-5p Enhances the Expression of the Cystic Fibrosis Transmembrane Conductance Regulator (CFTR) in Calu-3 Cells

**DOI:** 10.3390/molecules23010071

**Published:** 2017-12-29

**Authors:** Enrica Fabbri, Anna Tamanini, Tiziana Jakova, Jessica Gasparello, Alex Manicardi, Roberto Corradini, Giuseppe Sabbioni, Alessia Finotti, Monica Borgatti, Ilaria Lampronti, Silvia Munari, Maria Cristina Dechecchi, Giulio Cabrini, Roberto Gambari

**Affiliations:** 1 Department of Life Sciences and Biotechnology, University of Ferrara, 44121 Ferrara, Italy; enrica.fabbri@unife.it (E.F.); gspjsc@unife.it (J.G.); giuseppe.sabbioni@student.unife.it (G.S.); alessia.finotti@unife.it (A.F.); monica.borgatti@unife.it (M.B.); ilaria.lampronti@unife.it (I.L.); 2 Laboratory of Molecular Pathology, University-Hospital, 37126 Verona, Italy; anna.tamanini@aovr.veneto.it (A.T.); silvia.munari88@gmail.com (S.M.); cristina.dechecchi@ospedaleuniverona.it (M.C.D.); giulio.cabrini@univr.it (G.C.); 3 Department of Chemistry, Life Sciences and Environmental Sustainability, University of Parma, 43124 Parma, Italy; tiziana.jakova@studenti.unipr.it (T.J.); Alex.manicardi@ugent.be (A.M.); roberto.corradini@unipr.it (R.C.)

**Keywords:** peptide nucleic acids, cystic fibrosis, microRNAs, miR-145, miRNA targeting, delivery, CFTR

## Abstract

Peptide nucleic acids (PNAs) are very useful tools for gene regulation at different levels, but in particular in the last years their use for targeting microRNA (anti-miR PNAs) has provided impressive advancements. In this respect, microRNAs related to the repression of cystic fibrosis transmembrane conductance regulator (CFTR) gene, which is defective in cystic fibrosis, are of great importance in the development of new type of treatments. In this paper we propose the use of an anti-miR PNA for targeting miR-145, a microRNA reported to suppress CFTR expression. Octaarginine-anti-miR PNA conjugates were delivered to Calu-3 cells, exerting sequence dependent targeting of miR-145-5p. This allowed to enhance expression of the miR-145 regulated CFTR gene, analyzed at mRNA (RT-qPCR, Reverse Transcription quantitative Polymerase Chain Reaction) and CFTR protein (Western blotting) level.

## 1. Introduction

MicroRNAs (miRNAs, miRs) are from 19 to 25 nucleotide-long noncoding RNAs that regulate gene expression by targeting mRNAs, leading to a translational repression or mRNA degradation [1,2,3,4]. Since their discovery the number of microRNA sequences deposited in the miRBase databases has grown significantly [5]. The complex networks constituted by miRNAs and mRNAs lead to the control of highly regulated biological functions, such as differentiation, cell cycle and apoptosis [6].

Epigenetic regulation of expression of cystic fibrosis transmembrane conductance regulator (CFTR) gene by miRNAs has been recently explored by different groups in cystic fibrosis (CF) primary bronchial epithelial cells in vitro or from bronchial brushings ex vivo. Expression of miR-145 and miR-494 was found anti-regulated with that of CFTR [7]. The effect of air pollutants and cigarette smoke on CFTR expression identified two more miRNA that could target CFTR, namely miR-101 and miR-144 [8]. Genome-wide expression of miRNAs in primary non-CF bronchial epithelial cells revealed the expression of miR-138 as a down-regulator of SIN3A, a transcriptional repressor of CFTR [9]. A second study of miRNA profiling showed a high expression of miR-494 and miR-509-3p in the CF cells and a direct interaction with CFTR transcript [10]. These in vitro findings were confirmed and extended in ex vivo analyses which evidenced an increased expression of miR-494, miR-223 and miR-145 in CF brushings of airway cells [11]. Altogether, different miRNAs which have been found increased in CF primary bronchial epithelial cells can reduce CFTR expression, either by direct (miR-145, miR-223, miR-494, miR-509-3p) or indirect (miR-138) actions. Therefore, targeting miRNAs, such as miR-145, might be an important strategy for up-regulating CFTR.

Peptide nucleic acids (PNAs) are DNA analogues of outstanding properties [12,13,14], since, despite a radical structural change with respect to DNA and RNA, they are capable of sequence-specific and efficient hybridization with complementary DNA and RNA, forming Watson-Crick double helices [12]. In addition, they are able to generate triple helix formation with double stranded DNA and perform strand invasion [15,16]. Accordingly, they have been used as very efficient tools for pharmacologically-mediated alteration of gene expression, both in vitro and in vivo [17,18,19,20]. PNA and PNA-based analogues were proposed as antisense molecules targeting mRNAs, as triple-helix forming molecules targeting eukaryotic gene promoters, as artificial promoters, or as decoy molecules targeting transcription factors [16,17,18,19,20]. Recently, PNAs have been shown to be able of altering biological functions of microRNAs, both in vitro and in vivo [21,22,23,24,25,26,27,28]. Cheng et al., for instance, demonstrated that attachment of a peptide–(anti-miR)PNA conjugate is able to target the tumor microenvironment and to transport the anti-miR PNA across plasma membranes under acidic conditions such as those found in solid tumors. This treatment led to an efficient inhibition of the target oncomiR in a tumor mouse model [27].

The objective of this study was to design a PNA targeting miR-145, determine its activity in inhibiting miR-145, and verify whether it induces an increase of CFTR production. The PNA was conjugated to a poly-arginine tail, since this type of constructs was previously used by our group for the delivery of PNA into cell lines [24,25,26]. As experimental model system the Calu-3 cell line (American Type Culture Collection, ATCC HTB-55) has been selected [29,30]. These cells are a well-differentiated and characterized cell line derived from human bronchial submucosal glands and extensively used to study CFTR expression and immunological behavior [9,10,11,12].

## 2. Results

### 2.1. Location of miR-145-5p Binding Sites within the 3’-UTR Sequence of CFTR mRNA: Comparison of the miR-145/CFTR Interactions with Other miR-145 Regulated mRNAs

**Figure 1:**
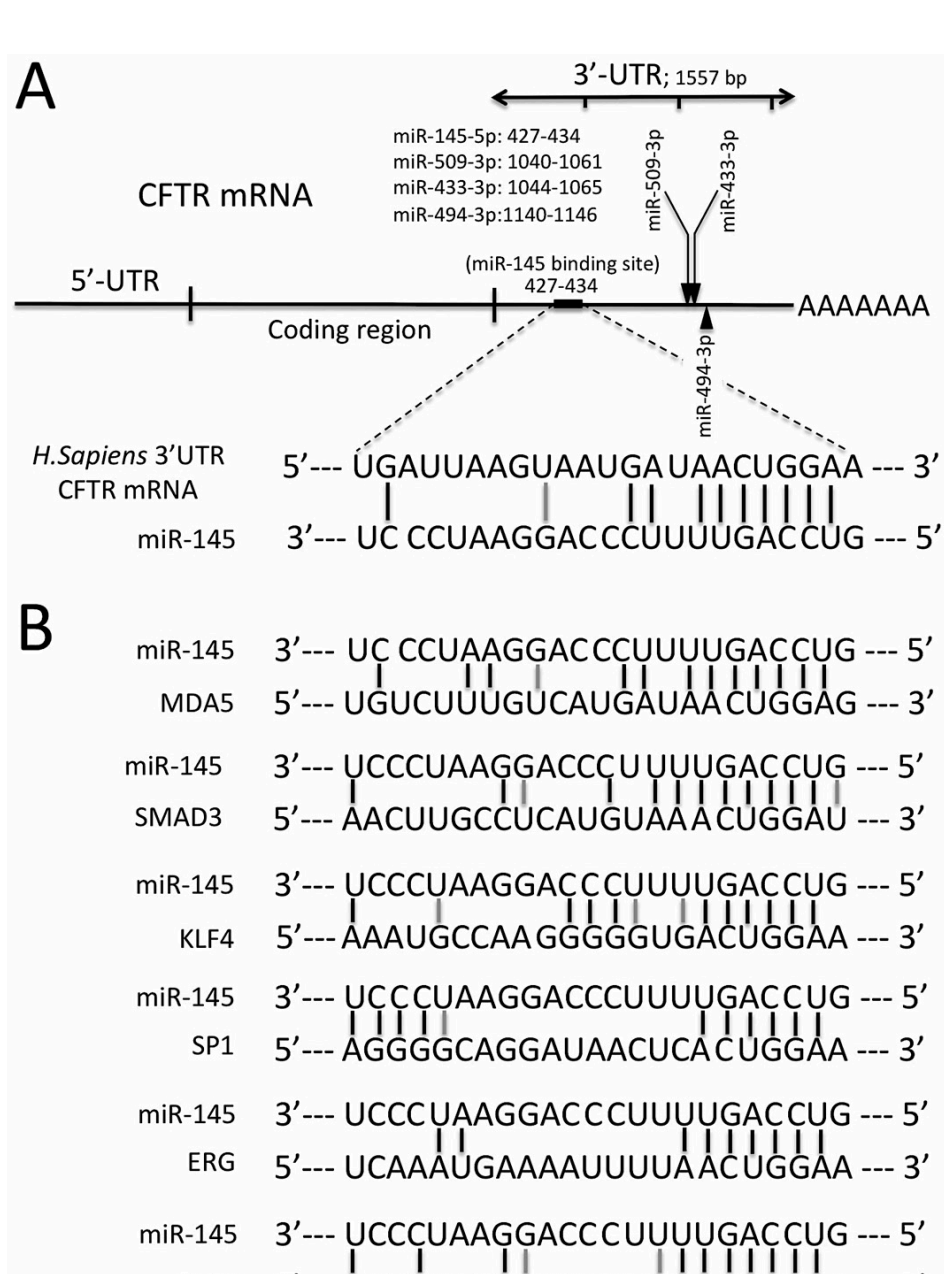
(**A**) Location of the miR-145-5p binding sites within the CFTR 3’-UTR region. (**B**) Interactions between miR-145 and validated mRNA targets: melanoma differentiation-associated protein 5 (MDA5) [33], SMAD family member 3 (SMAD3) [34], Kruppel-like factor 4 (KLF4) [35], specific protein Sp1 transcription factor (SP1) [36], ETS-related gene (ERG) [37] and cyclin dependent kinase 6 (CDK6) [36]. GC/CG and AT/TA: black lines; GU/UG: gray lines.

Figure 1A shows the location of the miR-145 binding sites within the 3’-UTR sequence (position 427–437 of the 1557 bp long 3’-UTR of the human CFTR mRNA) [10,11]. In addition, locations of the binding sites of miR-433, miR-509-3p and miR-494 (considered in this paper) are also shown [31,32].

In Figure 1A, the interaction between miR-145-5p and CFTR sequences is also shown. The number of CG/GC, AT/TA and GU/UG interactions is 11, a value similar or even higher to the number of possible interactions between miR-145 and other miR-145 regulated genes (see Figure 1B) [33,34,35,36,37]. The mRNAs considered have been validated as miR-145 regulated on the basis of luciferase reporter activity directed by 3’-UTR sequences carrying miR-145 binding sites and gene expression alterations using RT-qPCR (Reverse Transcription quantitative Polymerase Chain Reaction), Western blotting and, in some cases, functional assays [11,33,34,35,36,37]. These analyses confirm miR-145-5p as being e true regulator of CFTR gene expression.

### 2.2. MiR-145-5p Binding Sites of the 3’-UTR Sequence of CFTR Are Conserved throughout Evolution

**Figure 2:**
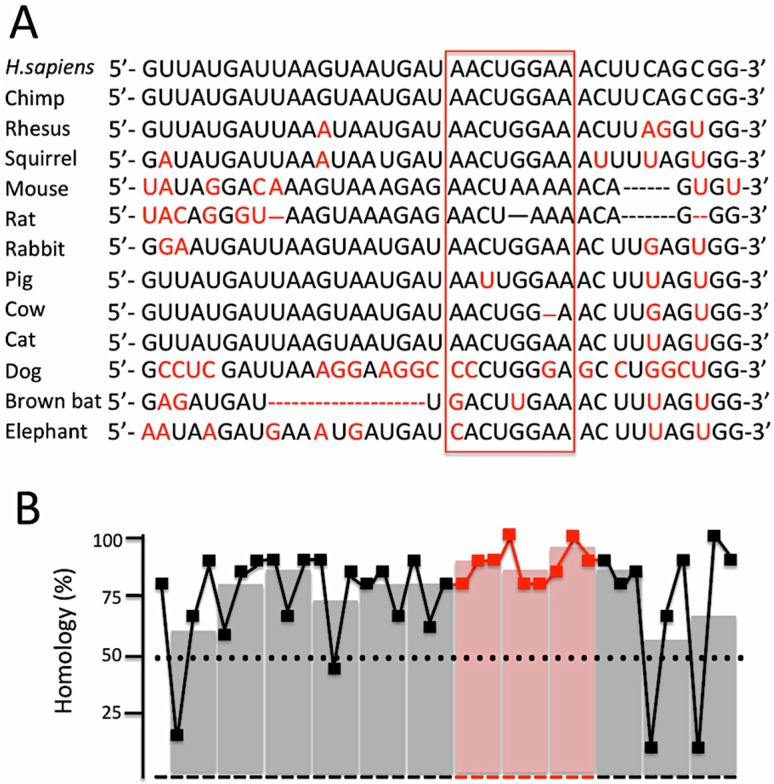
The binding sites for miR-145-5p are conserved throughout molecular evolution of the CFTR gene. (**A**) Alignment of the miR-145-5p binding sites of *Homo sapiens* (*H. sapiens*), *Pan troglodytes* (Chimp), *Macaca Mulatta* (Rhesus), *Saimiri sciureus* (Squirrel), *Mus musculus* (Mouse), *Rattus norvegicus* (Rat), *Oryctolagus cuniculus* (Rabbit), *Sus scrofa* (Pig), *Bos taurus* (Cow), *Felis catus* (Cat), *Canis lupus familiaris* (Dog), *Myotis lucifugus* (Brown bat) and *Loxodonta africana* (Elephant). In red the differences with respect to the *Homo sapiens* sequence are underlined. The CFTR sequence complementary to miR-145-5p seed region is boxed; (**B**) Level of homology shown for each nucleotide (black and red symbols) as well as for arbitrarily chosen triplets (gray and slight red bars).

Figure 2 shows that the miR-145 binding sequence is highly conserved through evolution, suggesting a role in CFTR expression and functions. In Figure 2A the alignment is shown of the miR-145-5p binding sites of *Homo sapiens* (*H. sapiens*), *Pan troglodytes* (Chimp), *Macaca Mulatta* (Rhesus), *Saimiri sciureus* (Squirrel), *Mus musculus* (Mouse), *Rattus norvegicus* (Rat), *Oryctolagus cuniculus* (Rabbit), *Sus scrofa* (Pig), *Bos taurus* (Cow), *Felis catus* (Cat), *Canis lupus familiaris* (Dog), *Myotis lucifugus* (Brown bat) and *Loxodonta africana* (Elephant). In red the differences with respect to the *Homo sapiens* sequence are underlined. The CFTR sequence complementary to miR-145-5p seed region is boxed. The region showing the highest level of homology (reaching 100% homology in some positions) belongs to the seed-complementary stretch, as also shown in Figure 2B, were the homology is shown for each nucleotide as well as for arbitrarily chosen triplets. Taken together the data shown in Figure 2 further sustain the concept of a role of miR-145-5p binding sites in CFTR regulation.

### 2.3. R8-PNA-a145 Inhibits miR-145-5p

**Figure 3:**
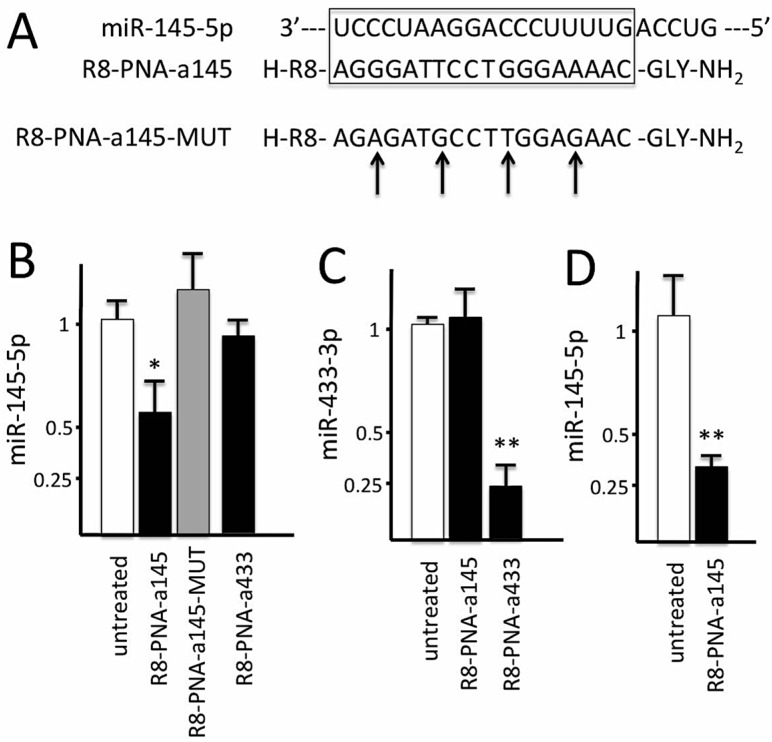
(**A**) Sequences of the R8-PNA-a145 and the mutated R8-PNA-a145-MUT. The G- > A, T- > G and A- > G mutations are arrowed. The box localizes the regions of full complementarity between miR-145-5p and R8-PNA-a145 sequences; (**B**,**C**). Inhibition of miR-145-5p (**B**) and miR-433-3p (**C**) hybridization signals in Calu-3 cells treated for 72 h with R8-PNA-a145 (**B**,**C**), mutated R8-PNA-a145-MUT (**B**) and R8-PNA-a433 (**B**,**C**) molecules (2 μM, as indicated); (**D**) Inhibition of miR-145-5p in NuLi-1 cells treated for 72 h with R8-PNA-a145 (2 μM). Results shown represent the average ± standard deviation (S.D.) obtained in at least three independent experiments. * = *p* < 0.05; ** = *p* < 0.01.

The PNAs were designed according to our standardized protocols, using the following criteria: (a) length of 18 bp, suitable for efficient synthesis also on large scale; (b) lack of self-complementarity both in antiparallel and parallel orientation; (c) minimal length of complementary sequences in mRNA, as evaluated by BLAST search; (d) when possible, targeting of the “seed region” which is an essential element for miRNA function. A carrier octaargine (R_8_) peptide was conjugated at N-terminus of the PNA chain since it induces an efficiency in the delivery which approaches 100% (i.e., uptake in 100% of the target cell population), as elsewhere published [24,25,26]; this conjugation is easily realized during PNA solid-phase synthesis using the same reagents and solvents. We have previously reported the higher efficiency of R_8_-PNA conjugates (R8-PNAs) in inhibiting target miRNAs when this activity is compared to that of conventional commercially available antagomiRNAs [38]. Control PNA (R8-PNA-a145-MUT) was obtained by modification of the position of four nucleobases, thus leaving the same base composition. The mutated sequence was also analyzed using BLAST search to assess possible interferences. Figure 3A reports the sequences of PNA selected in the present study. R8-PNA-a145-5p displays a fully complementary sequence with respect to R8-PNA-a145-MUT harboring three changes suppressing the hybridization as shown by circular dichroism (data not shown). HPLC-MS data characterizing the synthesized PNAs are presented in Figures S1–S5 in Supplementary Materials.

When Calu-3 cells were cultured in the presence of R8-PNA-a145 and of the mutated sequence R8-PNA-a145-MUT, a clear-cut result was obtained and depicted in Figure 3B. First of all, treatment of Calu-3 cells with R8-PNA-a145 leads to an inhibition of the miR-145-5p hybridization signals, compatible with decrease of free miR-145-5p content, and hence of its potential action, in treated cells; second, the mutant R8-PNA-a145-MUT displayed no inhibitory effects. The decrease of miR-145 hybridization signals following treatment R8-PNA-a145 is expected, in agreement with the effects of anti-miRNA PNAs elsewhere published [24,25,26,38]. In addition, we found that the effects are fairly specific; in fact, the results obtained demonstrate that hybridization specific for other miRNAs expressed in Calu-3 cells (for instance miR-433, miR-494 and miR-509) were unchanged following R8-PNA-a145 treatment. The representative experiments shown in Figure 3B,C demonstrate no effect of R8-PNA-a145 on miR-433-3p (Figure 3C) and, conversely, an inhibitory effect of R8-PNA-a433 on miR-433-3p (Figure 3C) with only minor effects on miR-145-5p (Figure 3B). Altogether these experiments support the concept that the effects of R8-PNA-a145 on miR-145-5p are sequence-specific. Finally, the effects of R8-PNA-a145 were also confirmed on other cell lines, such the lung epithelial NuLi-1 cell line (see the representative experiment shown in Figure 3D) [39].

### 2.4. Effects of the R8-PNA-a145 on CFTR

**Figure 4:**
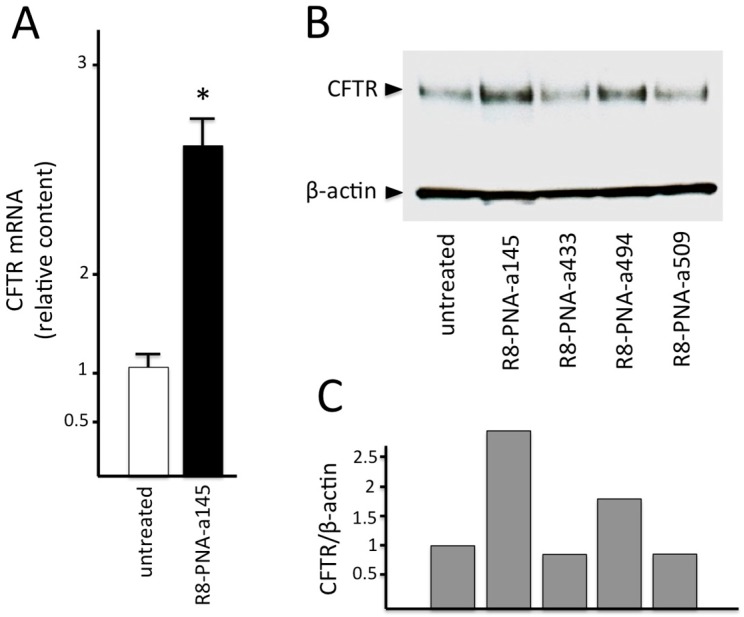
Upregulation of CFTR mRNA (RT-qPCR, (**A**); *N* = 3; * = *p* < 0.05) and CFTR protein (Western blotting, (**B**,**C**)) in Calu-3 cells cultured for 72 h in the absence or in the presence of R8-PNA-a145-5p. CFTR and β-actin is indicated in panel (**B**) with arrowheads. Effects of PNAs targeting miR-433, miR-509-3p and miR-494 are also shown in panel (**B**). In panel (**C**) the quantitation of the CFTR/β-actin ratios are shown and were obtained by densitometric analysis of the Western blotting shown in panel (**B**).

When Calu-3 cells were cultured in the presence of R8-PNA-a145 a clear effect was observed in CFTR mRNA accumulation. In fact the CFTR mRNA fold increases were 2.7/2.9 with respect to untreated Calu-3 cells. This value was obtained after determining CFTR mRNA content by RT-qPCR analysis (Figure 4A). These results prompted us to determine the CFTR content by Western blotting, as shown in Figure 4B,C. Calu-3 cells were treated for 72 h with R8-PNA-a145 and then proteins were separated by polyacrylamide gel electrophoresis and Western blotting analysis was performed using two antibodies, one specific for CFTR, the other for β-actin, used as an internal control. The CFTR protein increase was found to be 2–2.5 times more in Calu-3 extracts after R8-PNA-a145-5p treatment in three independent experiments. As shown in the representative experiment reported in Figure 4B,C, the efficiency of R8-PNA-a145 in CFTR up-regulation was similar to that found using R8-PNA-a494, while no effects were observed using R8-PNA-a433 and R8-PNA-a509. These results suggest that miR-145-5p targeting should be considered in the development of miRNA-therapeutic protocols for CFTR up-regulation.

### 2.5. Lack of Antiproliferative and Pro-Apoptotic Effects of R8-PNA-a145 on Calu-3 Cells

**Figure 5:**
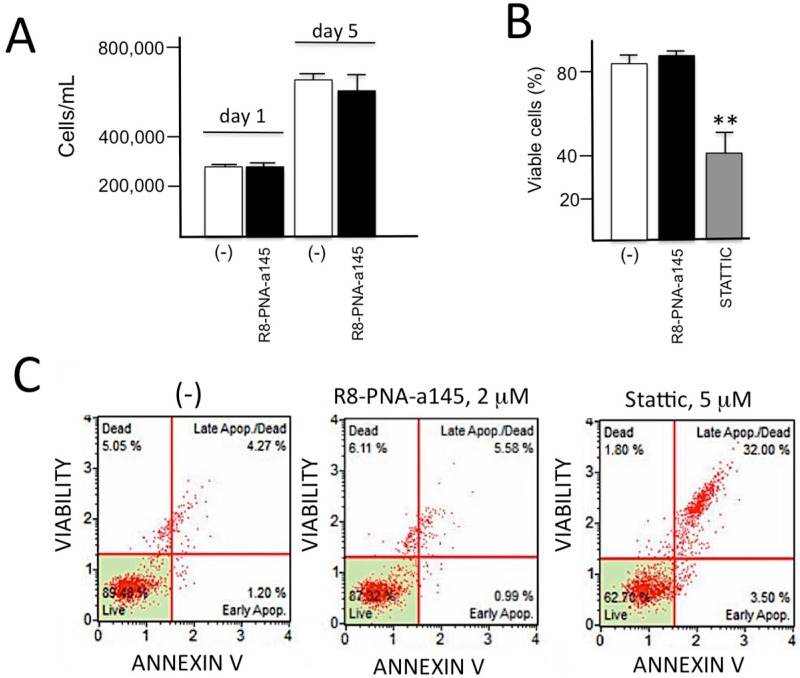
Effects of R8-PNA-a145 on cell growth, vitality and apoptosis. (**A**) Calu-3 cells were cultured in the absence or in the presence of R8-PNA-a145 different days and the cell number/mL determined; (**B**,**C**) Effects of R8-PNA-a145 on vitality (**B**) and apoptosis (**C**) of Calu-3 cells. Vitality was determined by trypan blue analysis of dead cells; apoptosis by the Annexin V & Dead Cell Kit (Millipore Corporation). Results in panels (**A**,**B**) represent the average ± S.D. of three independent experiments. ** = *p* < 0.01.

The results shown in Figure 5 were performed in order to verify whether R8-PNA-a145-5p was to some extent cytotoxic. Figure 5 shows that the studied PNA did not exhibit antiproliferative effects (panel A), did not reduce the extent of viable cells (panel B) and did not induce apoptosis when compared to the proapoptotic compound Stattic (panel C) [40]. In these experiments Calu-3 cells were cultured for 72 h in the absence, in the presence of R8-PNA-a145 or with the apoptotic inducers Stattic combined to 10% dimethyl sulfoxide (DMSO). After this treatment, the cell number/mL was determined, the dead cells were measured and the possible induction of apoptosis was detected. The Annexin V and Dead Cell assay was performed with “Muse” (Millipore Corporation, Billerica, MA, USA) method, according to the instructions supplied by the manufacturer. This procedure utilizes Annexin V to detect phosphatidylserine (PS) on the external membrane of apoptotic cells [26]. A dead cell marker is also used as an indicator of cell membrane structural integrity. It is excluded from live, healthy cells, as well as early apoptotic cells. Four populations of cells can be distinguished in this assay. Cells were washed with sterile PBS 1X, trypsinized, suspended and diluted (1:2) with the one step addition of the Muse Annexin V & Dead Cell reagent. After incubation of 20 min at room temperature in the dark, samples were analyzed. Data from prepared samples were acquired and recorded utilizing the Annexin V and Dead Cell Software Module (Millipore). The results obtained are shows in Figure 5C and demonstrate that apoptosis was not induced by PNAs targeting miR-145-5p, whereas under the same conditions an increase of apoptosis was observed using Stattic (Selective STAT3 inhibitor) with 10% DMSO.

## 3. Discussion

The data presented in this short report show that a PNA directed against miR-145-5p is able to inhibit miR-145-5p and increase the miR-145-5p regulated CFTR. The increase of CFTR expression was detectable at the level of mRNA (analyzed by RT-qPCR) and protein (analyzed by Western blotting). These data are of interest because are a proof-of-principle that miRNA targeting might be considered to increase CFTR content, with possible applications in the personalized therapy of cystic fibrosis. The field of precision medicine is in fact growing [41,42,43]. With respect to different molecular and genetic basis of CF, it is expected that miR-145 targeting will not be useful for CFTR defect of type I (no protein), II (no traffic), III (no function). On the contrary, increase of CFTR is expected to be useful for type IV (less function), V (less protein) and VI (less stable protein) CFTR defect [41,42,43]. In any case combined therapy using read-through molecules (for type I), splicing correctors (for type V) might be proposed.

**Figure 6:**
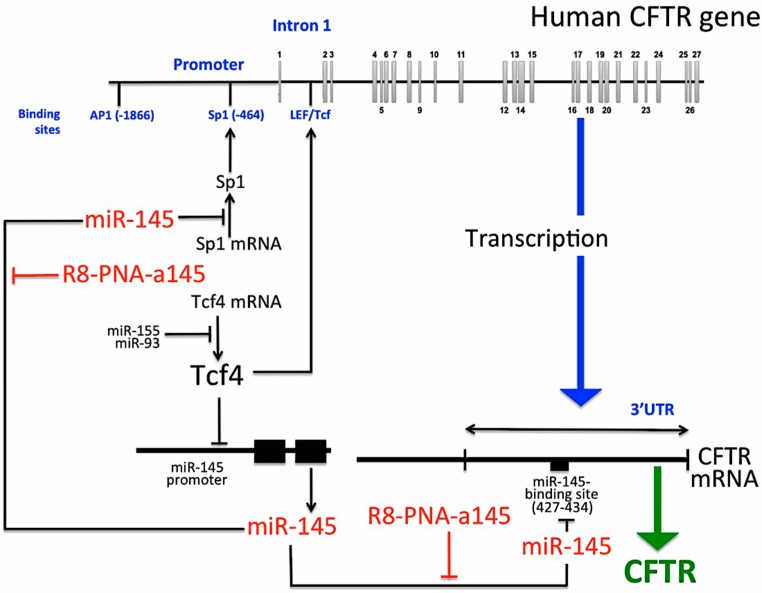
Proposed model for the interplay between transcription factors, CFTR regulation, miR-145-5p transcription and miR-145-5p target molecules.

In respect to miR-145-5p involvement in CFTR expression, we have to underline that the promoter of this microRNA is under a strong negative control, one of the involved transcription factors being Tcf4 [44]. Interestingly, Tcf4 binds to an intronic enhancer present in the Intron I of the CFTR gene (Figure 6) [45]. In this respect, the epigenetic signature of CFTR expression is coordinated via chromatin acetylation through a complex intronic element recognized by multiple transcription factors complexes, including AP2 (AP2-binding elements) and LEF/Tcf [45]. Therefore, low expression of Tcf4 (as it occurs in cystic fibrosis) [46,47,48] might lead to low transcription of the CFTR gene and high miR-145-5p dependent down modulation of CFTR mRNA. Therefore, a PNA targeting miR-145-5p might lead to upregulation of CFTR mRNA even in Tcf4 mediated high transcription level of the miR-145 gene cluster.

While the effects of the R8-PNA-a145 are specific and reproducible, we cannot exclude other biological effects of miR-145 leading to increased CFTR expression. For instance, several transcription factors have been reported to regulate CFTR transcription such as inverted CCAAT (Y box) binding proteins, CRE (cAMP-regulatory element) binding protein, Sp1 (specificity protein 1), AP (activator protein) 1 [46,47,48]. Among these, two important transcription factors interact with the CFTR promoter, AP1 and Sp1. In particular, in consideration of the fact that miR-145-5p down regulate Sp1 [36], the R8-PNA-a145 might also have an effect of Sp1 content regulating CFTR not only at the translational level (by interfering with the direct binding of miR-145-5p to the 3’-UTR CFTR sequences) but also at the transcriptional level by interfering with the miR-145-5p/Sp1 mRNA interactions.

For a possible translation to therapeutic approaches for CF, our data are just a proof-of-principle and presently limited in their application potential. In fact, concerning miRNA targeting we should consider that, in addition to miR-145-5p, several other miRNAs have been proposed to down-regulate CFTR expression, such as miR-494, miR-509-3p, miR-101, miR-433 [7,8,9,10,11,31,32]. Therefore, screening of PNAs targeting these miRNAs, identification of the most active and combined treatments using the more efficient should be considered to reach CFTR increase compatible with physiological effects. Moreover, our finding should be validated in primary CF cultures as well as in vivo model systems, to demonstrate that this approach can be potentially used in humans.

A final comment is related to the fact that miRNAs and transcription factors acts as complex networks. This might have important implications on drug design suggesting to develop approaches able to perform multi-targeting of microRNA involved in complex activities. For instance, given the central role of Tcf4 in enhancing CFTR transcription and inhibiting miR-145-5p expression, a possible therapeutic strategy might consider enhancing Tcf4 [44,45]. With respect to miRNA, considering that miR-155 are among the possible Tcf4 targeting miRNAs [49,50], the development of anti-miR-155 PNAs might be of interest also considering the target miRNAs are important modulators of other CFTR regulator such as FOXO1 [50]. The working hypothesis concerning the Tcf4/miR-145/CFTR network is depicted in Figure 6. In conclusion, miR-145-5p targeting for CFTR induction might be considered just one of the activities to be modulated to reach CFTR correction suitable for cystic fibrosis therapy.

## 4. Materials and Methods

### 4.1. Synthesis and Characterization of PNAs

The synthesis and characterization of anti-miR-145 PNAs was similar to those previously reported [51]. The synthesis was performed using standard Fmoc-based automate peptide synthesizer (Syro I, MultiSynTech GmbH, Witten, Germany), using a ChemMatrix-RinkAmide resin loaded with Fmoc-Gly-OH (0.2 mmol/g) as first monomer and using commercially available monomers (Link Technologies, Bellshill, UK) with HBTU/DIPEA coupling. Cleavage from the solid support was performed with 10% m-cresol in trifluoroacetic acid, followed by precipitation and washings with diethyl ether. Purification was performed by HPLC using a Jupiter RPC1 column (250–4.6 mm, 1.7 μM, Phenomenex, Torrance, CA, USA). Gradient: 100% A for 5 min, then from 0 to 50% B in 30 min at 0.25 mL/min flow (A: water +0.1% trifluoroacetic acid; B: acetonitrile +0.1% trifluoroacetic acid). Column temperature: 40 °C. After purification the PNAs were characterized by HRMS using the following HPLC-MS instrumental set-up: DIONEX Ultimate3000 system (Thermo Scientific, Waltham, MA, USA) coupled with a LTQ Orbitrap XL spectrometer (Thermo Fisher Scientific, Waltham, MA, USA); Software: Xcalibur 2.0.7 SP1 (Thermo Fisher Scientific). Column: AERIS Peptides 3.6 u XB-C18 150 × 2.1 mm (Phenomenex). Chromatographic condition: eluent A: water +0.2% formic acid; eluent B: CAN + 0.2% Formic acid. Column temperature: 35 °C. Program: initial isocratic at 10% B (5 min), then linear gradient to 95 % B (in 25 min). Final wash with 95% B for 10 min. Flow rate: 0.2 mL/min. Mass parameters:analyzer:FTMS; mass range: 200–2000 *m*/*z*.

The concentration of the PNA was calculated using UV-absorbance at 260 nm assuming an additive contribution of all bases (ε = 198,000 M^−1^ cm^−1^ for both R8-PNA-a145 and R8-PNA-a145-MUT). Characterisation of PNAs with HPLC-MS is reported in Supporting Information (Figures S1–S5).

***R8-PNA-a145*:** sequence H-R_8_-AGGGATTCCTGGGAAAAC-Gly-NH_2_; R_t_ = 11.55 min, HRMS: calculated *MW*: 6272.4698 g/mol; *m*/*z* found (calculated): 1569.71 (1569.84) [MH_4_]^4+^, 1255.97 (1256.07) [MH_5_]^5+^, 1046.81 (1046.9) [MH_6_]^6+^, 897.41 (897.48) [MH_7_]^7+^, 785.36 (785.42) [MH_8_]^8+^, 698.21 (698.26) [MH_9_]^9+^.

***R8-PNA-a145-MUT*:** sequence H-R_8_-AGAGATGCCTTGGAGAAC-Gly-NH_2_; R_t_ = 10.23 min, HRMS: calculated *MW*: 6272.4698 g/mol; *m*/*z* found (calculated): 1569.70 (1569.84) [MH_4_]^4+^, 1255.97 (1256.07) [MH_5_]^5+^, 1046.81 (1046.9) [MH_6_]^6+^, 897.41 (897.48) [MH_7_]^7+^, 785.36 (785.42) [MH_8_]^8+^, 698.21 (698.26) [MH_9_]^9+^.

***R8-PNA-a509*:** sequence H-R_8_-CTACCCACAGACGTACCA-Gly-NH_2_; R_t_ = 11.17 min, HRMS: calculated *MW*: 6097.4456 g/mol; *m*/*z* found (calculated): 1525.95 (1526.07) [MH_4_]^4+^, 1220.96 (1221.06) [MH_5_]^5+^, 1017.64 (1017.71) [MH_6_]^6+^, 872.40 (872.47) [MH_7_]^7+^, 763.48 (763.54) [MH_8_]^8+^, 678.76 (678.81) [MH_9_]^9+^, 610.98 (611.03) [MH_10_]^10+^

***R8-PNA-a494*:** sequence H-R_8_-TTTCCCGTGTATGTTTCA-Gly-NH2; R_t_ = 12.30 min, HRMS: calculated *MW*: 6146.4044 g/mol; *m*/*z* found (calculated): 1538.19 (1538.32) [MH_4_]^4+^, 1230.75 (1230.86) [MH_5_]^5+^, 1025.80 (1025.88) [MH_6_]^6+^, 879.40 (879.47) [MH_7_]^7+^, 769.60 (769.66) [MH_8_]^8+^, 684.20 (684.25) [MH_9_]^9+^, 615.88 (615.93)[MH_10_]^10+^

***R8-PNA-a433*:** sequence H-R_8_-CGGGGAACCCTTCAAGAT-Gly-NH2; R_t_ = 10.47 min, HRMS: calculated *MW*: 6208.4525 g/mol; *m*/*z* found (calculated): 1036.14 (1036.22) [MH_6_]^6+^, 888.26 (888.33) [MH_7_]^7+^, 777.35 (777.41) [MH_8_]^8+^, 691.09 (691.15) [MH_9_]^9+^.

### 4.2. Cell Lines and Culture Conditions

Calu-3 [29,30] and NuLi-1 cells [39] were cultured in humidified atmosphere of 5% CO_2_/air in DMEM/F12 medium (Gibco, Grand Island, NY, USA) supplemented with 10% fetal bovine serum (Biowest, Nauillè, France), 100 units/mL penicillin and 100 μg/mL streptomycin (Lonza, Verviers, Belgium) and 1% NEEA (100×) (Non-Essential Amino Acids Solution; Gibco, Thermo Fisher Scientific, Waltham, MA, USA). To determine the effect on proliferation, cell growth was monitored by determining the cell number/mL using a Z2 Coulter Counter (Coulter Electronics, Hialeah, FL, USA).

### 4.3. RNA Extraction

Cultured cells were trypsinized and collected by centrifugation at 1500 rpm for 10 min at 4 °C, washed with PBS, lysed with Tri-Reagent (Sigma Aldrich, St. Louis, MO, USA), according to manufacturer’s instructions. The isolated RNA was washed once with cold 75% ethanol, dried and dissolved in nuclease free pure water before use. RNA from human tissue sections was extracted and purified with miRNeasy FFPE Kit (Qiagen GmbH, Hilden, Germany) according to the manufacture's procedures. 

### 4.4. Quantitative Analyses of miRNAs

For miRNA quantification using real-time RT-qPCR reagents, the primers and probes (hsa-miR-145, TM:002278) were obtained from Applied Biosystems (Foster City, CA, USA). Reverse transcriptase (RT) reactions were performed using the TaqMan MicroRNA Reverse Transcription Kit (Applied Biosystems); real-time PCR was performed according to the manufacturer’s protocols [26]. The quantity of 300 ng per sample were used for the assays. All RT reactions, including no-template controls and RT-minus controls, were performed in duplicate using the CFX96 Touch Real-Time PCR Detection System (BioRad, Hercules, CA, USA). The relative expression was calculated using the comparative cycle threshold method and as reference U6 snRNA (TM:001973) and hsa-let-7c (TM:000379) were used to normalize all RNA samples, since it remains constant in the assayed samples by miR-profiling and quantitative RT-PCR analysis, as previously reported [24,26].

### 4.5. Analysis of CFTR Expression: RT-qPCR

Gene expression analysis was performed by RT-qPCR. 300 ng of the total RNA was reverse transcribed by using random hexamers. Quantitative real-time PCR (qPCR) assays were carried out using gene-specific double fluorescently labeled probes. Primers and probes used to assay CFTR (Assay ID: Hs00357011_m1) gene expression were purchased from were from Applied Biosystems, (Applied Biosystems). The relative expression was calculated using the comparative cycle threshold method and, as reference genes, the human RPL13A (Assay ID: Hs03043885_g1) [52,53]. 

### 4.6. Analysis of CFTR Expression: Western Blotting

Cell pellets were lysed in 1% Nonidet P40 (IGEPAL), 0.5% sodium deoxycholate, 200 mM NaCl, 10 mM Trizma base, pH 7.8, 1 mM EDTA plus protease inhibitor mixture and 1 mM PMSF for 30 min in ice. Lysates were cleared by centrifugation at 10,000× *g* for 10 min at 4 °C. Protein concentration was determined by the method of Lowry after precipitation with 5% Trichloroacetic acid (TCA), utilizing bovine serum albumin as a standard. For CFTR analysis 20 μg of total protein was heated in Laemmli buffer (Bio-Rad) at 37 °C for 10 min and loaded onto a 3 to 8% Tris-acetate gel (Bio-Rad). The gel proteins were transferred to PVDF membrane (Bio-Rad) by using Trans Blot Turbo (Bio-Rad) and processed for Western blotting by using mouse monoclonal antibody, clone 596, against NBD2 domain of CFTR (University of North Carolina, Cystic Fibrosis Center, Chapel Hill, NC, USA) at a dilution of 1:2500 by an overnight incubation at 4 °C. After washes, membranes were incubated with horseradish peroxidase-coupled anti-mouse immunoglobulin (R&D System, Minneapolis, MN, USA) at room temperature for 1 h and after washes the signal was developed by enhanced chemiluminescence (LumiGlo Reagent and Peroxide, Cell Signaling). After membranes stripping, β-Actin monoclonal antibody (Sigma-Aldrich) was used to confirm the equal loading of samples [54]. 

### 4.7. Analysis of Apoptosis

Annexin V and Dead Cell assay on Calu-3 cells, untreated and treated for 72 h with 2 μM PNA-a145, 5 μM Stattic together with 10% DMSO, were performed with “Muse” (Millipore Corporation, Billerica, MA, USA) method, according to the instructions supplied by the manufacturer. This procedure utilizes Annexin V to detect PS (PhosphatidylSerine) on the external membrane of apoptotic cells. A dead cell marker is also used as an indicator of cell membrane structural integrity. It is excluded from live, healthy cells, as well as early apoptotic cells. Four populations of cells can be distinguished in this assay. Cells were washed with sterile PBS 1X, trypsinized, suspended and diluted (1:2) with the one step addition of the Muse Annexin V & Dead Cell reagent. After incubation of 10 min at room temperature in the dark, samples were put on ice, vortexed and analyzed. Data from prepared samples are acquired and recorded utilizing the Annexin V and Dead Cell Software Module (Millipore) [36].

### 4.8. Statistical Analysis

Results are expressed as mean ± standard deviation (S.D.) Comparisons between groups were made by using paired Student’s *t* test. Statistical significance was defined with *p* < 0.05 (*, significant) and *p* < 0.01 (**; highly significant).

## Supplementary Materials

The supplementary materials are available online.

## Abbreviations

CFTR	cystic fibrosis transmembrane conductance regulator
PNA	peptide nucleic acid
RT-qPCR	reverse transcription quantitative polymerase-chain reaction
