# Supplementary material for: A Peptide Nucleic Acid against MicroRNA miR-145-5p Enhances the Expression of the Cystic Fibrosis Transmembrane Conductance Regulator (CFTR) in Calu-3 Cells
Source: Molecules. 2017 Dec 29;23(1):71. doi: 10.3390/molecules23010071 (PMC6017273; doi:10.3390/molecules23010071)
Supplement: Supplementary file 1 [file molecules-23-00071-s001.pdf]

Supplementary Materials

A Peptide Nucleic Acid against MicroRNA miR-145-5p Enhances the Expression of the Cystic Fibrosis Transmembrane Conductance Regulator (CFTR) in Calu-3 Cells

Enrica Fabbri [1], Anna Tamanini [2], Tiziana Jakova [3], Jessica Gasparello [1], Alex Manicardi [3,†], Roberto Corradini [3], Giuseppe Sabbioni [1], Alessia Finotti [1], Monica Borgatti [1], Ilaria Lampronti [1], Silvia Munari [2], Maria Cristina Dechecchi [2], Giulio Cabrini [2] and Roberto Gambari [1,*]

Index

Figure S1. HPLC MS analysis of *R8-PNA-a145*

Figure S2. HPLC MS analysis of *R8-PNA-a145-MUT*

Figure S3. HPLC MS analysis of *R8-PNA-a509*

Figure S4. HPLC MS analysis of *R8-PNA-a494*

Figure S5. HPLC MS analysis of *R8-PNA-a433*

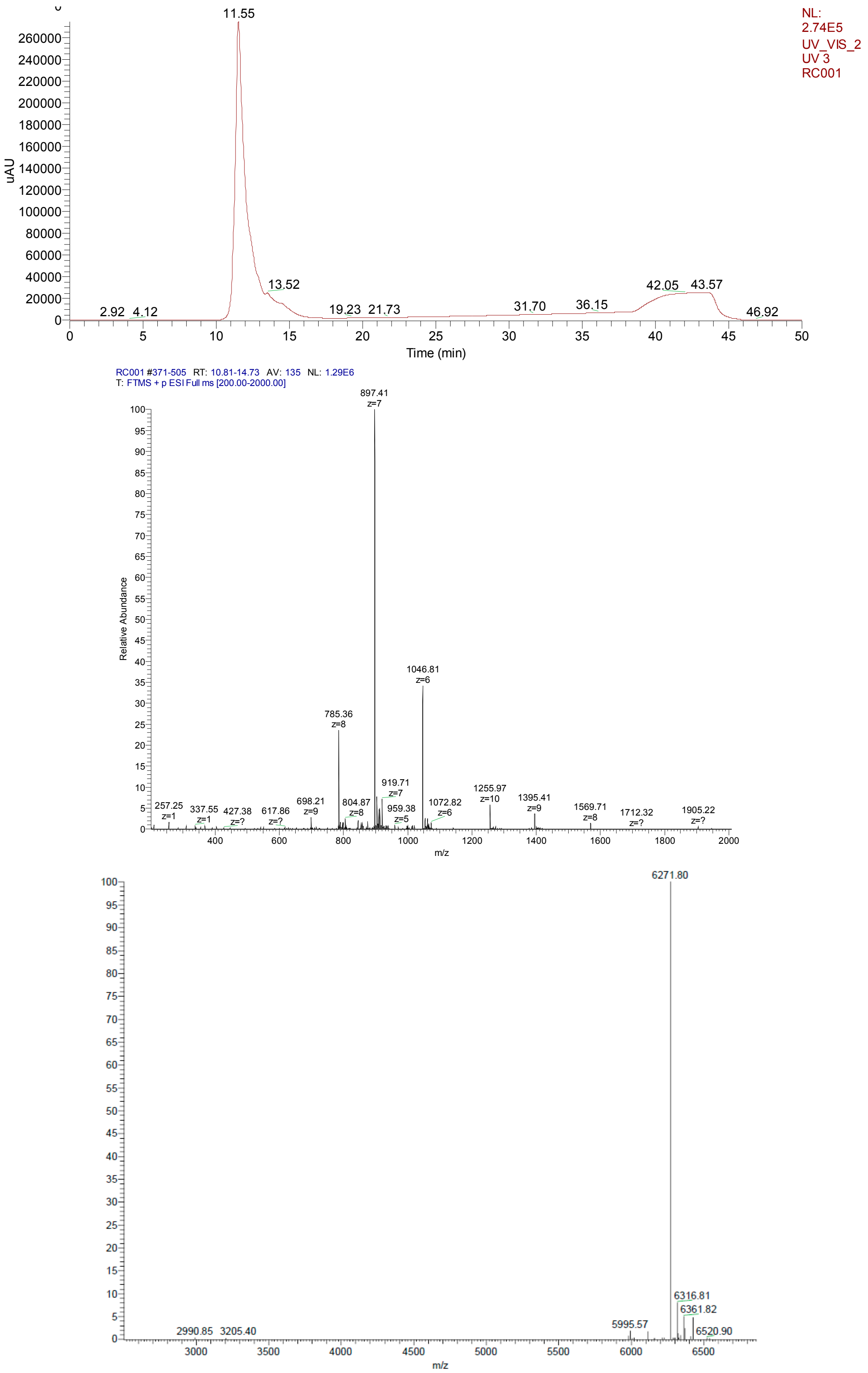

Figure S1. HPLC-HRMS (Orbitrap) analysis of *R8-PNA-a145:* above HPLC chromatogram, middle: ESI-MS spectrum of peak at 11.55 min; below: mathematical deconvolution of the multicharged signals. Conditions are as indicated in the Materials and Methods part.

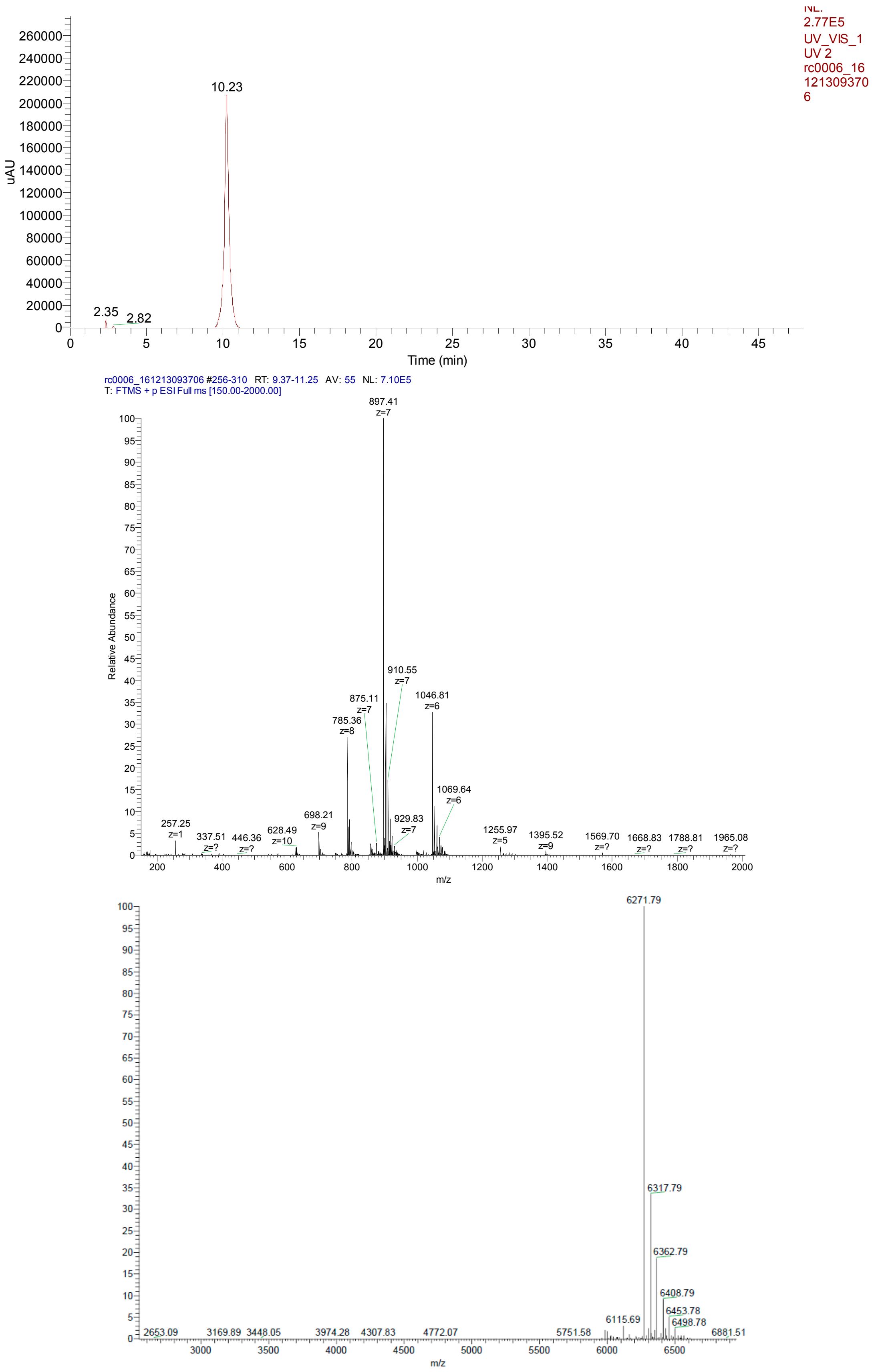

Figure S2. HPLC-HRMS (Orbitrap) analysis of *R8-PNA-a145-Mut:* above HPLC chromatogram (UV detector, 260 nm), middle: ESI-MS spectrum of peak at 11.55 min; below: mathematical deconvolution of the multicharged signals. Conditions are as indicated in the Materials and Methods part.

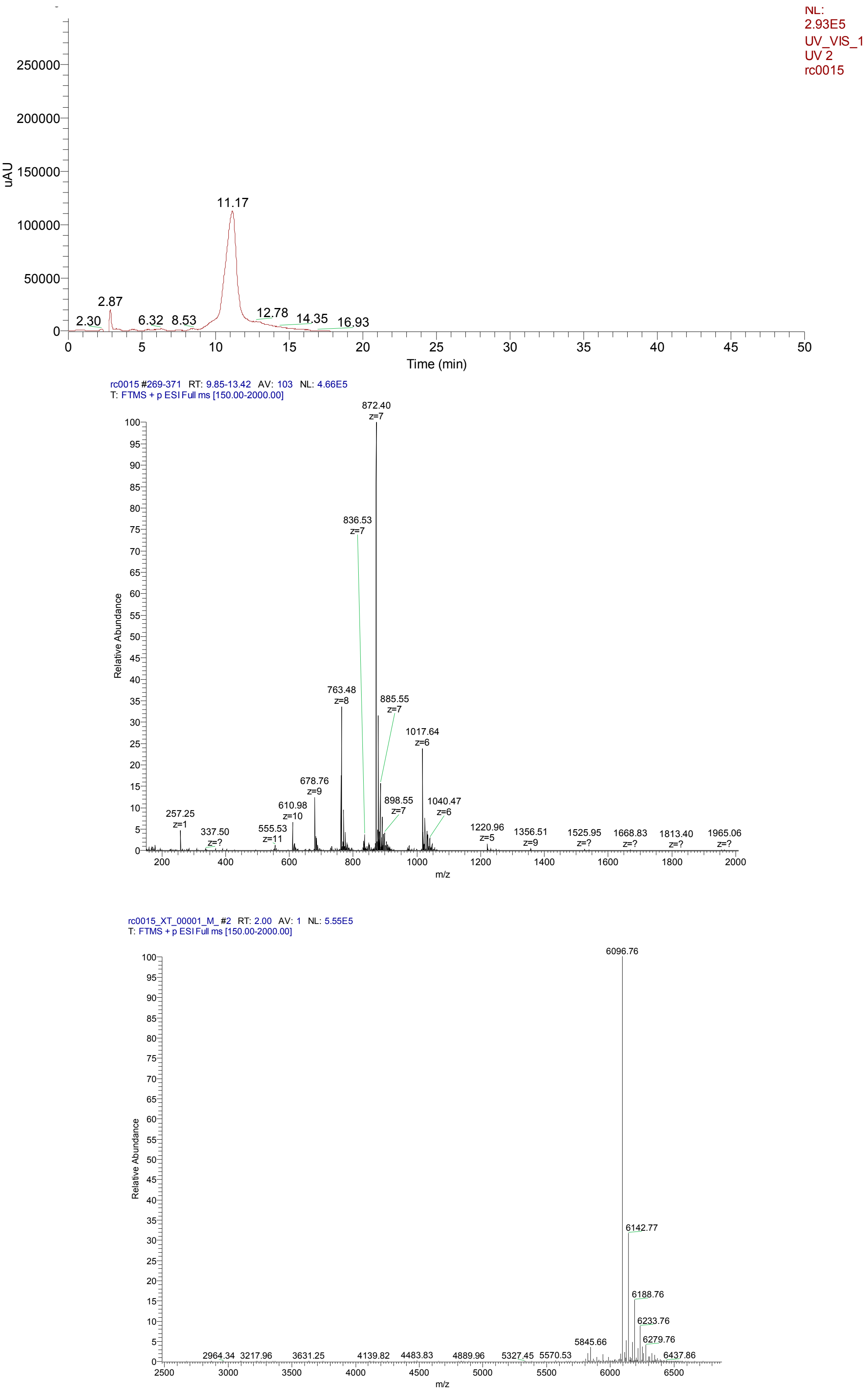

Figure S3. HPLC-HRMS (Orbitrap) analysis of *R8-PNA-a509:* above HPLC chromatogram, middle: ESI-MS spectrum of peak at 11.55 min; below: mathematical deconvolution of the multicharged signals. Conditions are as indicated in the Materials and Methods part.

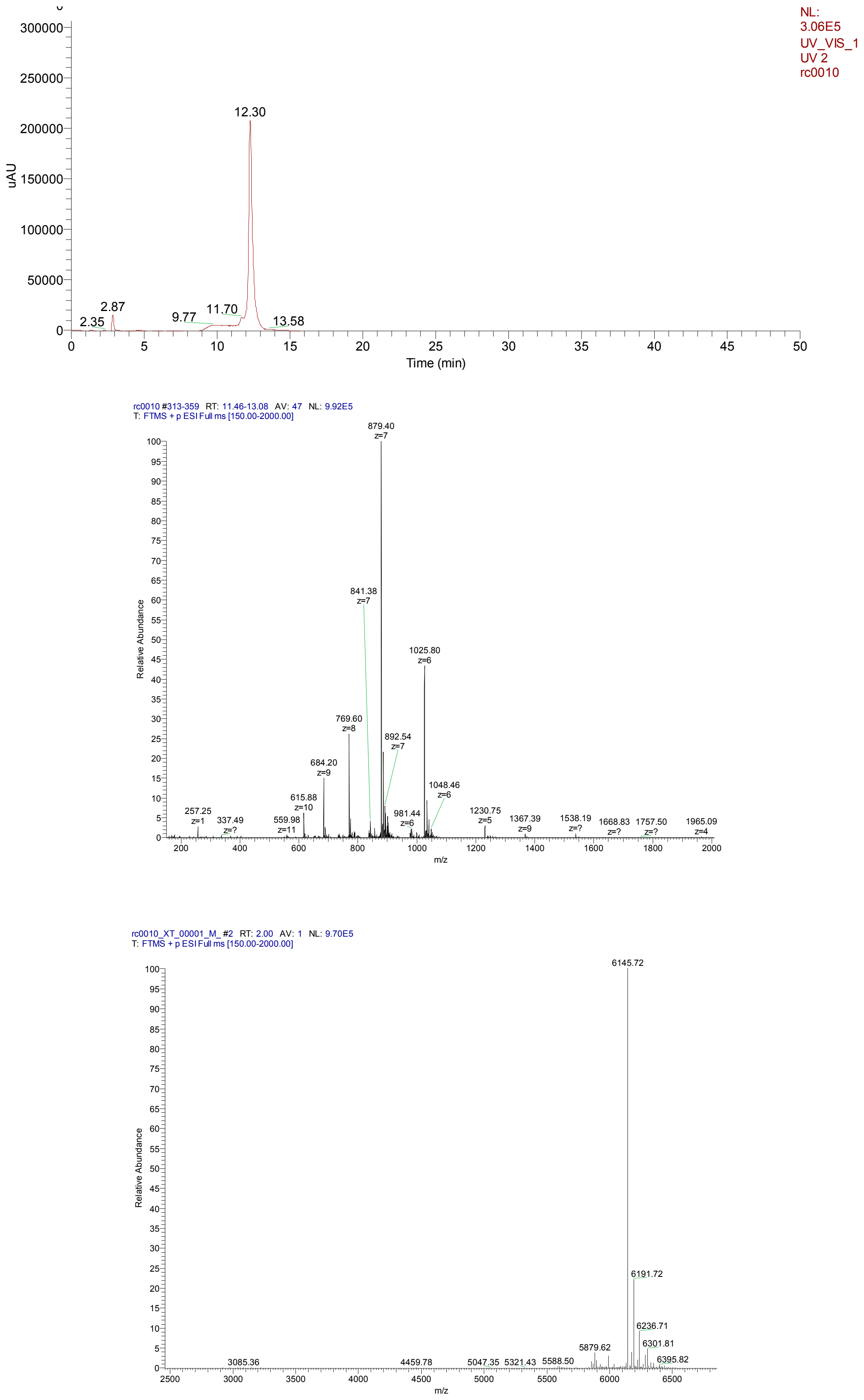

Figure S4. HPLC-HRMS (Orbitrap) analysis of *R8-PNA-a494:* above HPLC chromatogram, middle: ESI-MS spectrum of peak at 11.55 min; below: mathematical deconvolution of the multicharged signals. Conditions are as indicated in the Materials and Methods part.

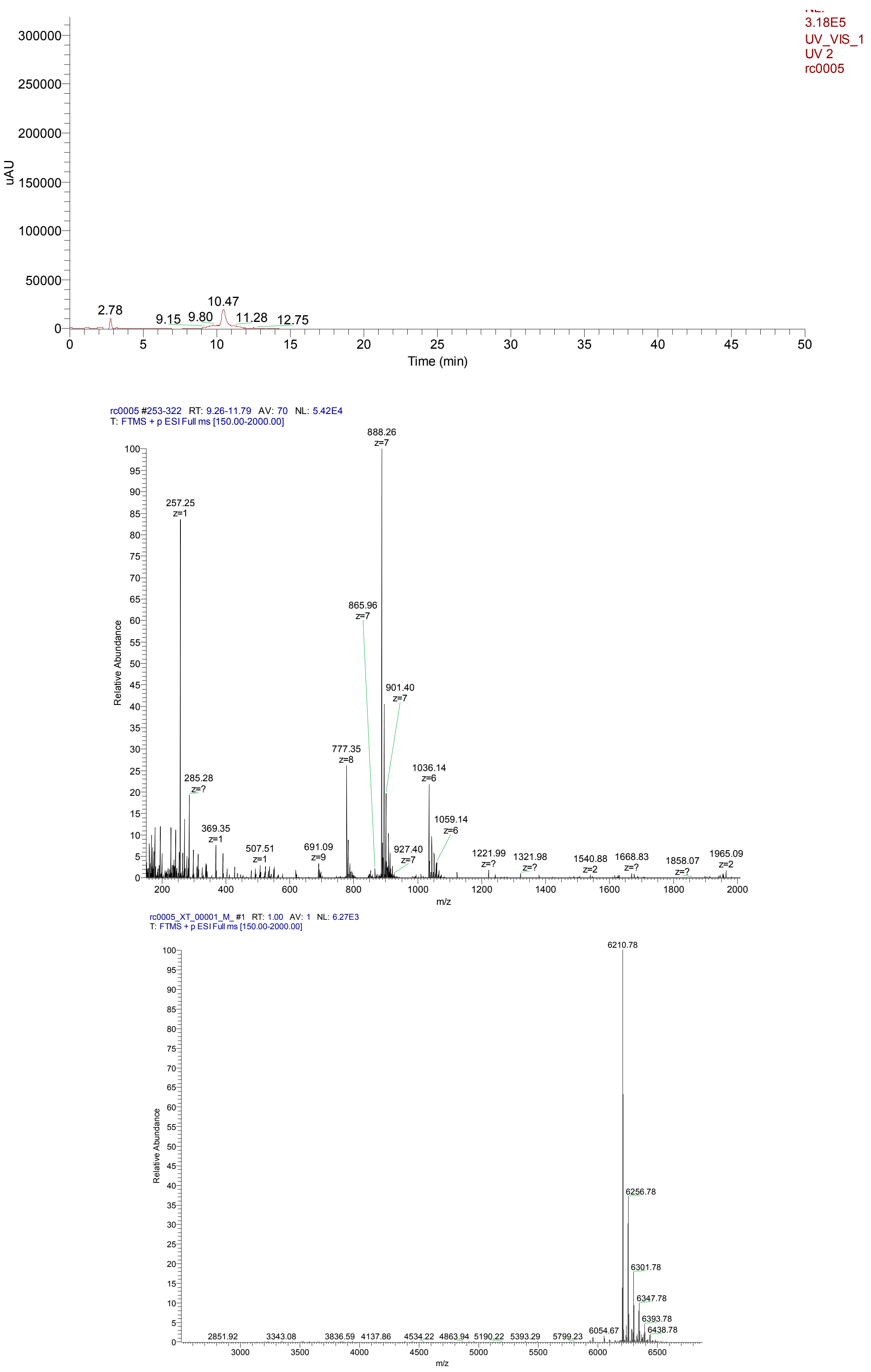

Figure S5. HPLC-HRMS (Orbitrap) analysis of *R8-PNA-a433:* above HPLC chromatogram, middle: ESI-MS spectrum of peak at 11.55 min; below: mathematical deconvolution of the multicharged signals. Conditions are as indicated in the Materials and Methods part.
